# Validation of Cross-Genotype Neutralization by Hepatitis B Virus-Specific Monoclonal Antibodies by *In Vitro* and *In Vivo* Infection

**DOI:** 10.1371/journal.pone.0118062

**Published:** 2015-02-18

**Authors:** Susumu Hamada-Tsutsumi, Etsuko Iio, Tsunamasa Watanabe, Shuko Murakami, Masanori Isogawa, Sayuki Iijima, Takako Inoue, Kayoko Matsunami, Kazuto Tajiri, Tatsuhiko Ozawa, Hiroyuki Kishi, Atsushi Muraguchi, Takashi Joh, Yasuhito Tanaka

**Affiliations:** 1 Department of Virology and Liver Unit, Nagoya City University Graduate School of Medical Sciences, Nagoya, Japan; 2 Department of Gastroenterology and Metabolism, Nagoya City University Graduate School of Medical Sciences, Nagoya, Japan; 3 The Third Department of Internal Medicine, Graduate School of Medical and Pharmaceutical Sciences, University of Toyama, Toyama, Japan; 4 The Department of Immunology, Graduate School of Medical and Pharmaceutical Sciences, University of Toyama, Toyama, Japan; Kobe University, JAPAN

## Abstract

Vaccines based on hepatitis B virus (HBV) genotype A have been used worldwide for immunoprophylaxis and are thought to prevent infections by non-A HBV strains effectively, whereas, vaccines generated from genotype C have been used in several Asian countries, including Japan and Korea, where HBV genotype C is prevalent. However, acute hepatitis B caused by HBV genotype A infection has been increasing in Japan and little is known about the efficacy of immunization with genotype C-based vaccines against non-C infection. We have isolated human monoclonal antibodies (mAbs) from individuals who were immunized with the genotype C-based vaccine. In this study, the efficacies of these two mAbs, HB0116 and HB0478, were analyzed using in vivo and in vitro models of HBV infection. Intravenous inoculation of HBV genotype C into chimeric mice with human hepatocytes resulted in the establishment of HBV infection after five weeks, whereas preincubation of the inocula with HB0116 or HB0478 protected chimeric mice from genotype C infection completely. Interestingly, both HB0116 and HB0478 were found to block completely genotype A infection. Moreover, infection by a genotype C strain with an immune escape substitution of amino acid 145 in the hepatitis B surface protein was also completely inhibited by incubation with HB0478. Finally, in vitro analysis of dose dependency revealed that the amounts of HB0478 required for complete protection against genotype C and genotype A infection were 5.5 mIU and 55 mIU, respectively. These results suggested that genotype C-based vaccines have ability to induce cross-genotype immunity against HBV infection.

## Introduction

Hepatitis B virus (HBV) is a blood-borne, hepatotropic virus that infects an estimated 350 million people worldwide. Besides the manifestations associated with acute hepatitis, chronic HBV infection constitutes a significantly high risk for the development of liver cirrhosis and hepatocellular carcinoma. HBV strains are classified into eight genotypes based on genetic diversity [1,2] and the prevalence of these genotypes varies geographically [3]. Hepatitis B surface antigen (HBsAg) is the key molecule for HBV entry into the hepatocyte [4] and HBV vaccination establishes host immunity by activating B lymphocytes that produce HBsAg-specific antibodies (anti-HBs) with neutralizing activities. The highly immunogenic region of HBsAg, known as the “a” determinant, comprises two peptide loops in which several amino acids vary among the HBV genotypes [5].

Vaccination of high risk individuals and universal infant/childhood vaccination programs have effectively decreased the incidence of acute HBV infection and consequent chronic hepatitis B [6]. Recombinant vaccines containing HBsAg generated from HBV genotype A2 (gt-A2) have been used worldwide. Although these A2-type vaccines are effective in preventing non-A2 HBV infections [7], investigation of cross-genotype protection is limited in the clinical setting. On the other hand, genotype B (gt-B) and genotype C (gt-C) strains are the most prevalent in east Asian countries [1] and some of these countries, including Japan and Korea, have used recombinant vaccines generated from gt-C for immunoprophylaxis against HBV endemic in these communities [8,9]. In the last decade, however, the spread of gt-A strains imported from foreign countries and the subsequent increase of hepatitis caused by HBV gt-A is a growing concern in Japan [10]. Until now, little is known about whether the gt-C HBV vaccine can induce effective immunity against non-C HBV infection.

Previously, we isolated human monoclonal antibodies (mAbs) against HBV from healthy volunteers who had been immunized with a gt-C type recombinant HBV vaccine (Biimugen), using a cell-microarray system [11–13]. A subsequent report revealed that among these mAbs, HB0116 and HB0478, recognize the first N-terminal peptide loop within the “a” determinant and have HBV-neutralizing activities [14]. In this report, whether these mAbs generated by the gt-C type vaccine can protect gt-A strain infections was investigated using in vitro and in vivo HBV infection models, including primary human hepatocytes (PHHs) and severe combined immunodeficient mice transgenic for urokinase-type plasminogen activator, whose livers were repopulated with human hepatocytes (hereafter referred to as chimeric mice) [15–17]. The neutralizing activities of these mAbs against the frequently isolated immune escape mutant, which has an amino acid substitution of arginine for glycine at residue 145 within the second, C-terminal loop of HBsAg (G145R) [18–20], were also investigated.

## Materials and Methods

### Ethics statement

This study conformed to the ethics guidelines of the 1975 Declaration of Helsinki as reflected by approval by the Ethics Committee of University of Toyama with written informed consent (Permit Number: 14–123). All animal experiments were carried out in strict accordance with the recommendations in the Guide for the Care Use of Laboratory Animals of the National Institute of Health. The animal protocol was approved by the Ethics Committees of PhoenixBio Co., Ltd (Permit Number: 0253). Chimeric mice were housed in specific pathogen—free facilities at the laboratory of PhoenixBio Co., Ltd. Food and water were delivered ad libitum. Chimeric mice were weighed and anesthetized using isofluorane prior to blood collection from the orbital vein. The chimeric mice were anesthetized using isofluorane and sacrificed by exsanguination from the heart at the end of the experiment.

### HBV-specific mAbs and recombinant peptides

Recombinant HB0116 and HB0478 in IgG form were generated as described previously [14]. Synthetic peptides for the first loop of HBsAg gt-C and gt-A (123–137 gt-C: TCTIPAQGTSMFPSC; 123–137 gt-A: TCTTPAQGNSMFPSC) were generated also as described previously [14].

The binding activity of each mAb for recombinant peptides was examined by ELISA with streptavidin-coated plates (Nunc, Roskilde, Denmark). Plates were coated with the peptides at 10 μg/mL and nonspecific binding was blocked with PBS containing 3% bovine serum albumin (BSA). Each mAb was added to the wells for 2 hours, followed by washing and reaction with alkaline phosphatase-conjugated anti-human IgG (Sigma, Saint Louis, MO). The O.D. value at 405 nm was evaluated after addition of phosphate substrate (Sigma). Control human monoclonal IgG1 (cIgG, Athens Research & Technology, Athens, GA) was added at the same concentration as the control.

### Immunoprecipitation assay

1 × 10^4^ copies of HBV of gt-C, gt-A and G145R (gt-C with an amino acid substitution of arginine for glycine at position 145 of HBsAg) were incubated with 1 μg of mAbs diluted in 2% BSA/PBS or cIgG on a rotating wheel overnight at 4°C and then protein A-Sepharose beads (GE Healthcare) were added to the mixture and incubated for a further 4 hours. The beads were centrifuged briefly to remove the supernatants, washed four times with 1 mL 2% BSA/PBS and resuspended in 30 μL sample loading buffer (Tris/HCl (pH 6.8), 2% SDS, 5% 2-mercaptoethanol, 10% glycerol, 0·001% bromophenol blue). After boiling for 5 minutes, 15 μL aliquots were applied to 15% SDS-PAGE and the proteins were separated and transferred to a nitrocellulose membrane. HBsAg was detected using 1 μg/mL of a HB0116/HB0478 mixture, followed by anti-human IgG conjugates of horseradish peroxidase (1:5000, Sigma) as the secondary antibody. The bands were visualized with enhanced chemiluminescence (Amersham Biosciences, Buckinghamshire, UK).

### HBV-neutralizing assay using HepaRG cells

The HBV-neutralizing capacities of HB0116 and HB0478 were investigated using the HepaRG cell line (supplied by Biopredic International, Rennes, France). The HepaRG cells were cultured and differentiated as described previously [21,22]. 1 × 10^4^ copies of HBV and 1 μg of each mAb were preincubated for 1 hour at room temperature and then added to HepaRG cells in medium containing 4% polyethylene glycol (PEG) 8000 (Sigma-Aldrich, St. Louis, MO, USA). After overnight incubation, the HepaRG cells were washed gently three times with medium and then cultured with fresh medium. On day 7 after infection, cellular DNA was extracted and HBV DNA was quantified as described previously [14].

### In vivo HBV-neutralizing assay using chimeric mice

The chimeric mice were purchased from PhoenixBio Co, Ltd (Hiroshima, Japan). The HBV inocula used in this experiment were prepared as follows: culture supernatants from cells transfected with plasmids expressing HBV gt-C, gt-A, and G145R contained immature HBV virions [16] and chimeric mice were inoculated with these culture supernatants to obtain the monoclonal and intact infectious virions. After establishing viremia in these mice, the sera were collected and used as inocula after titration in another experimental chimeric mouse.

Firstly, 1x10^4^ copies of the sera of chimeric mice infected with gt-A, gt-C, G145R were incubated at 37°C for 2 hours in the presence of HB0116 and/or HB0478 and injected intravenously into chimeric mice. Five weeks after injection, serum HBV DNA was measured by quantitative polymerase chain reaction (PCR) as reported previously [23].

### In vitro HBV-neutralizing assay using PHHs isolated from chimeric mice

Freshly isolated PHHs were purchased from PhoenixBio Co., Ltd (Higashihiroshima, Japan). Briefly, human hepatocytes were collected from the livers of chimeric mice by collagenase perfusion and plated on collagen-coated 96-well multiplates at a density of 6.7 × 10^4^ cells per well. The cells were then grown in dHCGM medium (Dulbecco’s modified Eagle medium supplemented with 10% fetal bovine serum, 1 μg/mL of penicillin, 1 μg/mL of streptomycin, 20 mM HEPES, 15 μg/mL of L-proline, 0.25 μg/mL of human recombinant insulin, 50 nM dexamethazone, 5 ng/mL of human recombinant epidermal growth factor, 0.1 mM ascorbic acid, and 2% DMSO).

To investigate HBV kinetics, PHHs were inoculated with serum from HBV gt-C chimeric mice at 5 genomes per cell for 24 hours in the presence of 4% PEG 8000. The sera from chimeric mice contained excess subviral particles including HBs proteins. The cells were then washed three times with the medium to remove the inoculum, and the culture supernatants were collected and replenished with fresh medium on 2, 3, 5, 7, and 12 days post infection (dpi).

To optimize the infectious condition for the analysis of antibody neutralization, HBV gt-C at 10, 3, 1, and 0.3 genomes per cell was preincubated with or without 100 mIU of hepatitis B immune globulin (HBIG) for 2 hours and PHHs were inoculated with the HBV-HBIG mixture for 24 hours with PEG or for 48 hours without PEG. The cells were washed and the supernatants were collected as described above.

Antibody neutralization experiments were performed as follows. HBV gt-C or gt-A inocula at 10 genomes per cells (6.7 × 10^5^ genomes/well) were preincubated with 670, 67, 6.7, or 0.67 ng of HB0478 (corresponding to 550, 55, 5.5, or 0.55 mIU) and exposed to PHHs for 48 hours without PEG. The cells were then washed and the supernatants were collected as described above.

### Southern blot analysis of HBV DNA

Southern blot analysis was performed with full-length probes for HBV as described previously [24].

### Quantification of HBV DNA, pregenomic RNA and HBsAg

Total RNA and total DNA were extracted from PHHs using ISOGEN (Nippon Gene Co. Ltd., Tokyo, Japan) and SMITEST EX R&D Kit (Genome Science Laboratories, Tokyo, Japan), respectively. Purified total RNA was then reverse-transcribed using a High Capacity RNA-to-cDNA Kit (Applied Biosystems, Foster City, CA) according to the manufacturer’s instructions. Extracellular HBV DNA, intracellular HBV DNA and pregenomic RNA were quantified by real-time quantitative PCR using StepOne Plus and TaqMan Universal PCR Master Mix (Applied Biosystems, Foster City, CA). The samples were denatured by incubating for 10 minutes at 95°C and amplified for 45 cycles (95°C 15 seconds, 60°C 60 seconds) with specific primers and TaqMan fluorescent probes. HBV DNA was amplified using primers HBV-F (5′-CACATCAGGATTCCTAGGACC-3′), HBV-R (5′-AGGTTGGTGAGTGATTGGAG-3′), and TaqMan probe HBV-FT (5′-FAM-CAGAGTCTAGACTCGTGGTGGACTTC-TAMRA-3′). Primers HBV-PC-F (5′-GGTCTGCGCACCAGCACC-3′), HBV-DN-R (5′-GGAAAGAAGTCAGAAGGCAA-3′) and TaqMan probe HBV-FM (5′-FAM-TCCAAGCTGTGCCTT-MGB-3′) specifically amplify cDNA from precore RNA. Primers HBV-PG-F (5′-CACCTCTGCCTAATCATC-3′), HBV-DN-R and TaqMan probe HBV-FM amplifies cDNA from both precore RNA and pregenomic RNA. The amount of pregenomic RNA was calculated by subtracting the copy number of precore RNA amplification from that of precore/pregenomic RNA amplification [25]. Extracellular HBsAg was quantified by automated ELISA (Fujirebio Inc., Tokyo, Japan). The detection limits are 2 × 103 copies for HBV DNA, 2 × 102 copies for pregenomic RNA and 0.005 IU/mL for HBsAg.

## Results

### Influence of genotype and amino acid substitutions on recognition by HBV-specific mAbs

**Fig 1:**
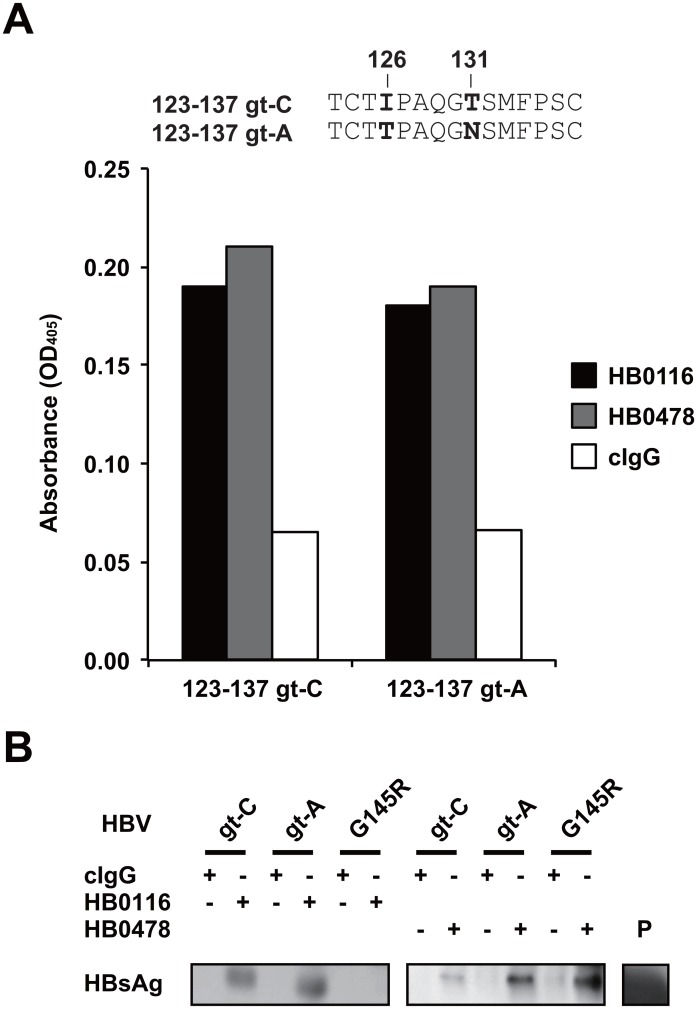
Binding capacity of mAbs HB0116 and HB0478 against with gt-C and gt-A HBsAg and the G145R variant. **(A)** Binding of mAbs HB0116 and HB0478 to synthetic peptides covering the first external loop of small-HBsAg was demonstrated by ELISA. The sequences of the recombinant peptides used in the analysis are shown above: amino acids which vary between genotype C (gt-C) and genotype A (gt-A) are indicated in bold. The absorbance at 405 nm is shown on the Y axis. Average data of three independent experiments are shown. **(B)** The gt-C, gt-A, and G145R virions were immunoprecipitated with HB0116 or HB0478 and HBsAg in the precipitates was detected by Western blotting. Recombinant HBsAg protein was used as the positive control (P lane). Representative data of three independent experiments are shown.

The mAbs HB0116 and HB0478 bind to the first loop (amino acids 123–137) of the “a” determinant and strongly inhibit HBV gt-C infection [14]. Therefore, whether the binding capacity of each mAb is affected by amino acid variation within the first loop was examined using recombinant peptides; there is amino acid variation between genotypes C and A at positions 126 (gt-C: I, gt-A: T) and 131 (gt-C: T, gt-A: N). Both HB0116 and HB0478 bound peptides not only corresponding to the first loop with the gt-C sequence but also corresponding to those with the gt-A sequence, indicating their cross-genotype recognition on binding in vitro (Fig. 1A). The binding capacities to the native HBs proteins of gt-A, gt-C, together with gt-C with the substitution G145R located within the second loop of HBsAg extracellular domain, were also examined. Interestingly, immunoprecipitation assays revealed that HB0116 bound to HBsAg of HBV gt-C and gt-A, but not to G145R, whereas HB0478 could bind to all three proteins (Fig. 1B).

**Fig 2:**
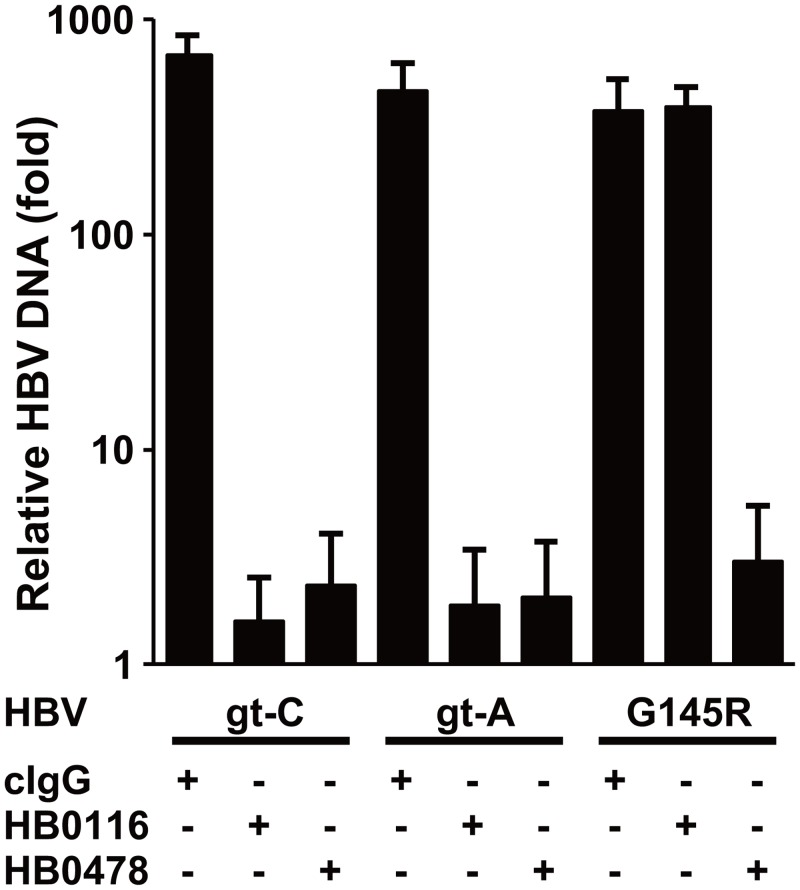
Relative HBV DNA concentrations in the total DNA extracted from HepaRG cells at 7 days after HBV infection. The Y-axis depicts the relative HBV DNA concentrations in the cells, with the concentrations on day 1 set at 1. Mean ± SD of three independent experiments are shown. cIgG, control human monoclonal IgG.

Next, the HBV-neutralizing activity of these mAbs was evaluated using HepaRG cells, which support HBV infection, by inoculating them with a high dose of HBV. Fig. 2 shows that HB0116 suppressed the increase of HBV DNA after inoculation of both HBV gt-C and gt-A, but could not inhibit infection by G145R. However, HB0478 could prevent infection by HBV gt-C, gt-A, and also G145R. These results are consistent with the immunoprecipitation results shown in Fig. 1B and indicate that HB0478 can bind to the first loop, regardless of genotype, and also bind to the G145R substituted protein, which is seen as an antibody escape variant in clinical practice.

### HB0116 and HB0478 protect against HBV gt-C and gt-A infections but only HB0478 protects against G145R mutant infection in vivo

The in vivo neutralizing activity of the mAbs was investigated using chimeric mice with human hepatocytes. After 1 × 10^4^ copies of HBV gt-C or gt-A were incubated with HB0116 and/or HB0478, the mixtures were injected intravenously into naïve chimeric mice and serum HBV DNA concentrations were measured for the evaluation of HBV infection at five weeks after injection. Although HBV gt-C infection was confirmed in the control experiment (Group 1, 9.8 × 10^3^ and 1.1 × 10^4^ copies/ml) (Table 1), preincubation of the inoculum with either 1 μg or 10 μg of HB0116 or HB0478 completely blocked HBV infection with both gt-C and gt-A (Groups 2–5 for gt-C, Groups 6–9 for gt-A). Meanwhile, inoculation of the HBV G145R strain into naïve chimeric mice resulted in the establishment of infection (Group 10, 1.0 × 10^4^ and 1.4 × 10^4^ copies/ml) and incubation with 10 μg of HB0116 had no impact on infection by G145R (Group 11, 1.1 × 10^4^–4.4 × 10^4^ copies/ml), whereas as little as 1 μg of HB0478 completely blocked G145R infection (Groups 12 and 15). Apparently, a combination of HB0116 and HB0478, either at 1 μg or 10 μg protected the chimeric mice from HBV infection (Groups 13 and 14).

**Table 1:** In vivo neutralization of HBV infection by monoclonal antibodies (mAbs).

HBV genotype	Group	HB0116 (μg/body)	HB0478 (μg/body)	HBV DNA (copies/mL)
C	Group 1	-	-	9.8 × 10^3^
	(n = 2)	-	-	1.1 × 10^4^
	Group 2	1	-	n.d.
	(n = 3)	1	-	n.d.
		1	-	n.d.
	Group 3	10	-	n.d.
	(n = 3)	10	-	n.d.
		10	-	n.d.
	Group 4	-	1	n.d.
	(n = 3)	-	1	n.d.
		-	1	n.d.
	Group 5	-	10	n.d.
	(n = 3)	-	10	n.d.
		-	10	n.d.
A	Group 6	1	-	n.d.
	(n = 3)	1	-	n.d.
		1	-	n.d.
	Group 7	10	-	n.d.
	(n = 3)	10	-	n.d.
		10	-	n.d.
	Group 8	-	1	n.d.
	(n = 3)	-	1	n.d.
		-	1	n.d.
	Group 9	-	10	n.d.
	(n = 2)	-	10	n.d.
G145R	Group 10	-	-	1.4 × 10^4^
	(n = 2)	-	-	1.0 × 10^4^
	Group 11	10	-	4.4 × 10^4^
	(n = 3)	10	-	1.1 × 10^4^
		10	-	3.3 × 10^4^
	Group 12	-	10	n.d.
	(n = 3)	-	10	n.d.
		-	10	n.d.
	Group 13	1	1	n.d.
	(n = 2)	1	1	n.d.
	Group 14	10	10	n.d.
	(n = 2)	10	10	n.d.
	Group 15	-	1	n.d.
	(n = 2)	-	1	n.d.

n.d.: not detected.

### Evaluation of PHHs isolated from chimeric mice with human hepatocytes as an in vitro HBV infection model

**Fig 3:**
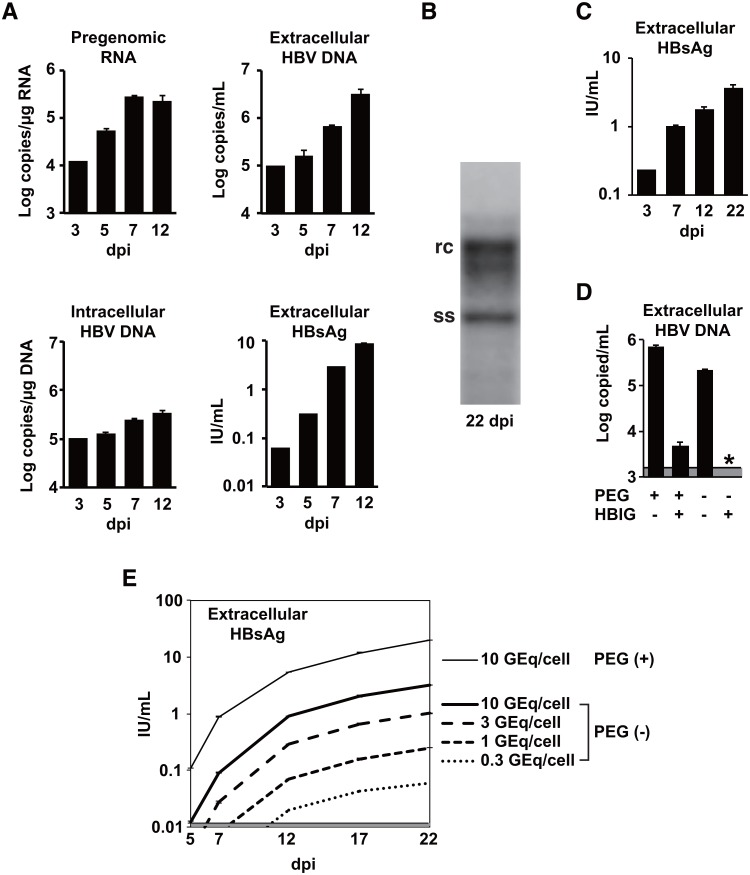
In vitro HBV infection model using PHHs isolated from chimeric mice with human hepatocytes. **(A)** PHHs were inoculated with HBV gt-C at 5 genomes per cell in the presence of PEG and intracellular pregenomic RNA, intracellular HBV DNA, extracellular HBV DNA and extracellular HBsAg were monitored by real-time quantitative PCR, or by automated ELISA. dpi, days post infection. **(B)** 20 μg of total DNA was extracted from PHHs 22 days after infection with HBV and analyzed by Southern blotting. Single-stranded HBV DNA (ss), a replication intermediate, and relaxed circular HBV DNA (rc) were detected. **(C)** Freshly prepared PHHs were inoculated with the day 52 supernatant from other HBV-infected PHHs. HBsAg secretion was monitored. **(D)** The use of PEG on HBV infection could mask the specificity of neutralization of HBV infection. Residual HBV infection was observed when PHHs were inoculated with a mixture of HBV and HBIG in the presence of PEG. An asterisk indicates a value below detection limit. **(E)** The efficacy of HBV infection without PEG was proportional to the size of the inoculum.

PHHs isolated from the chimeric mice with human hepatocytes were used to characterize further the neutralizing activity of mAb HB0478. In vitro HBV infection of the PHHs was confirmed by inoculating HBV gt-C at 5 HBV genomes per cell in the presence of 4% PEG 8000. The levels of pregenomic RNA, intracellular HBV DNA, extracellular HBV DNA, and extracellular HBsAg were monitored and it was found that all these viral products gradually increased from 3 to 12 dpi (Fig. 3A). Southern blot analysis of cell lysates revealed the presence of single-stranded HBV DNA as a replication intermediate in the infected PHHs, confirming HBV replication in the cells (Fig. 3B). Furthermore, culture supernatants from HBV-infected donor PHHs were inoculated into newly prepared PHHs. An increase of HBsAg production from the PHHs was observed following exposure of the cells to another culture supernatant containing HBV DNA (Fig. 3C), indicating that the donor PHHs produced infectious HBV virions (also known as Dane particles).

Next, to investigate whether this model can be adapted for the study of neutralizing activities against HBV infection, the effect of HBIG on HBV infection was evaluated in vitro. Fig. 3D shows that HBIG strongly reduced HBV infection but residual infection was detected in the presence of PEG, whereas, in the absence of PEG, the HBV infection was completely blocked by HBIG. These results indicate that, when neutralizing activities against HBV infection were investigated using this PHH system, inoculation without PEG is appropriate for the specificity of the establishment of HBV infection. However, because inoculation without PEG would be less efficient for HBV infection, the efficacy of HBV infection in the absence of PEG was also examined. Various titers of HBV (10, 3, 1, and 0.3 genomes per cell) were inoculated into PHHs and the HBsAg titers in the supernatants were monitored for 22 days (Fig. 3E). Although the HBsAg levels from PHHs infected without PEG were lower than those with PEG, the HBsAg levels in the supernatants were well correlated with the initial input of HBV (10 to 0.3 genomes per cells) in the absence of PEG. These results suggest that, albeit with somewhat lower infectivity, inoculation without PEG is available for neutralization assays using the PHH system.

### HB0478 efficiently blocks HBV infection by both gt-C and gt-A

**Fig 4:**
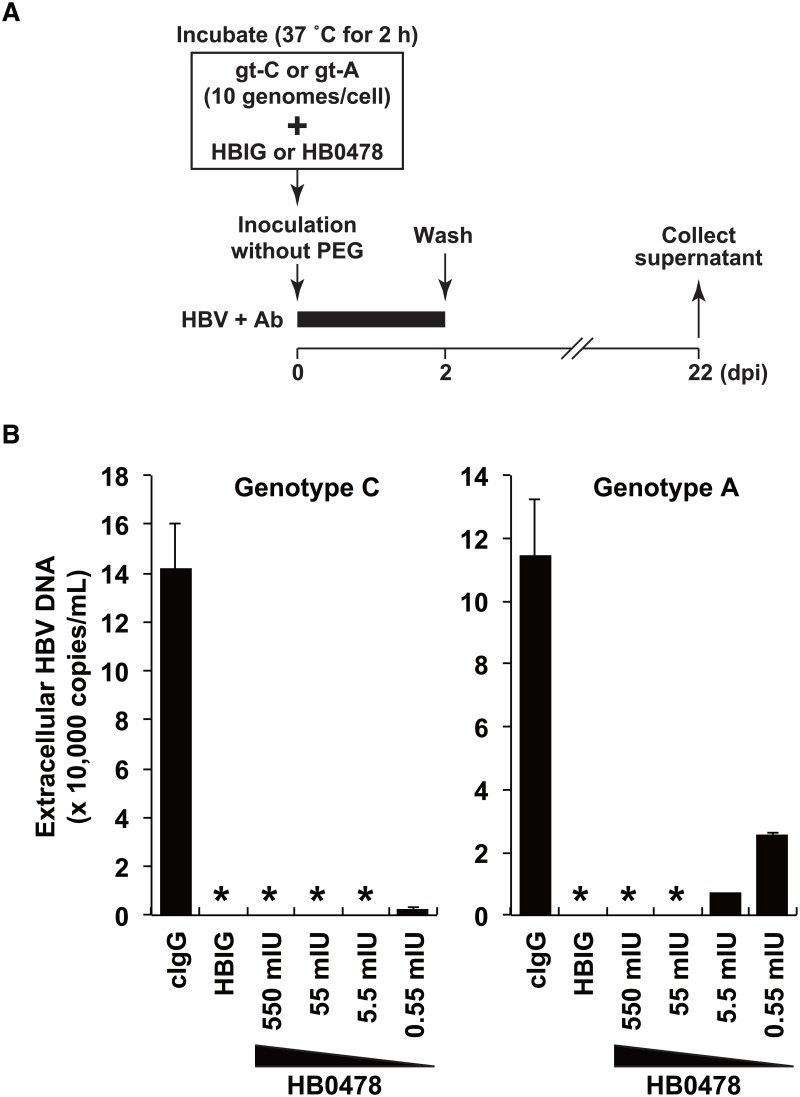
Titration of neutralization of gt-C and gt-A infection by mAb HB0478. HBV gt-C and gt-A were preincubated for 2 hours with 670 ng of control human IgG (cIgG), 100 mIU of HBIG, or 670, 67, 6.7 or 0.67 ng HB0478 (corresponding to 550, 55, 5.5, and 0.55 mIU) and PHHs were inoculated with the products at 10 genomes per cell. The Y-axis depicts the levels of extracellular HBV DNA in the supernatant harvested on 12 days post infection. Asterisks indicate values under the detection limit.

To evaluate the neutralizing activity of HB0478 against HBV infection, various amounts of HB0478 were preincubated with HBV gt-C or gt-A at 10 HBV genomes per cell (6.7 × 10^5^ genomes/well) for 2 hours and exposed to PHHs for 48 hours without PEG (Fig. 4A). Fig. 4B shows the levels of HBV DNA in the supernatants harvested at 22 dpi. HB0478 in the amounts of 550 and 55 mIU completely blocked the infection by both gt-C and gt-A (HBV DNA was never detected in the supernatant). 5.5 mIU of HB0478 also completely inhibited gt-C infection, while it strongly reduced but did not completely inhibit gt-A infection. These results indicate that mAb HB0478 has powerful neutralizing activity against HBV infection and that HB0478 generated by the gt-C type vaccine could protect against HBV infection by both gt-C and gt-A, although less effectively against gt-A.

## Discussion

Although the HBV vaccine strain used predominantly worldwide is genotype A2, genotype C strains are prevalent in Japan, where a selective vaccination program for high risk individuals with a gt-C-based vaccine is ongoing. A potential problem is that genotype A2 has been increasing recently as a cause of acute hepatitis B in Japan [10] and little is known about the efficacy of the gt-C-based vaccine against non-C HBV infection. In this report, we demonstrated that two mAbs, HB0478 and HB0116, derived from individuals immunized with the gt-C vaccine (Biimugen) that has been approved in Japan, neutralized HBV infections by both gt-C and gt-A in vitro and in vivo, suggesting that immunization with the gt-C vaccine could prevent infection by non-C HBV strains.

Epidemiological studies have shown that, in countries operating universal childhood vaccination programs using the gt-A2 vaccine, vertical transfer and/or incident infection of non-A2 were prevented efficiently [7]. Some studies have produced data supporting cross-genotype protection by immunization. An analysis of 221 mAbs isolated from volunteer HB vaccinees showed that 97% of them recognized common epitopes shared by all HBV genotypes [5]. The C(K/R)TC motif (amino acids 121–124), located in the N-terminal portion of the first loop of the “a” determinant of HBsAg, is conserved among all HBV genotypes (except for residue 122, K or R determining the serological subtype d or y, respectively) and highly immunogenic [26]. Moreover, a single mouse monoclonal Ab protected chimpanzees from infection by both adr (gt-C) and ayw (genotype D) strains [27].

Along with these findings, our results showed that the mAbs HB0478 and HB0116, generated following immunization with the gt-C type vaccine, neutralized the infectivity of both gt-C and gt-A HBV. In vitro experiments investigating dose dependency using freshly isolated PHHs also demonstrated that HB0478, at doses above 55 mIU, completely protected against both gt-C and gt-A infection, whereas HB0478, at a lower dose, 5.5 mIU, protected against gt-C infection only. It has been reported that analysis of nine HBV DNA positive blood donors in the United States revealed that 5 individuals who had been immunized with an A2-type vaccine were not protected against infections by non-A2 HBV [28]; however, the serum anti-HBs levels of these individuals (3–96 mIU/mL) were relatively low. Interestingly, the infections remained at a subclinical level in these vaccines, who subsequently resolved the HBV infection, suggesting that gt-A2 vaccination could not prevent non-A2 infection but can inhibit the development of clinical manifestations [28]. Therefore, it is possible that HBV specific antibodies, induced by gt-C vaccines, might be able to protect against clinical hepatitis caused by infection with non-C genotypes, even with lower anti-HBs concentrations. Further investigations are needed to determine clinical effectiveness of gt-C vaccine to induce cross-genotype immune responses.

Meanwhile, virus strains with amino acid substitutions in HBsAg often escape from HB vaccine-induced antibody and HBIG treatment during vertical transmission of HBV [19,20,29]. The substitution reported most frequently is residue 145, glycine to arginine (G145R), located in the second loop of the “a” determinant of HBsAg. This study demonstrated that HB0478 also recognized HBsAg with the G145R substitution and protected against G145R infection in vivo, whereas HB0116 did not bind to the G145R substituted protein or neutralize the mutant. Although how G145R in the second loop affects mAb-binding to the first loop is largely unknown, it is possible that the C(K/R)TC-dependent HB0478 epitope might be more distant from the second loop than that of HB0116, suggesting that HB0478 might not be affected by the conformational change of HBsAg induced by substitution of glycine at residue 145. It is noted that epitopes other than “a” determinant such as those within pre-S2 region [30] could also contributed to the neutralization of escape mutants.

In conclusion, this study raises the possibility that active immunization with a gt-C-based vaccine confers prophylaxis against gt-A, which is spreading in Japan, and against escape mutants such as G145R, when the anti-HBs responses are sufficient. Note that PHHs isolated from chimeric mice with human hepatocytes enabled us to investigate precisely the inhibitory effects of the mAbs, or any antiviral compounds, against HBV infection in vitro.
